# Resazurin Reduction-Based Assays Revisited: Guidelines for Accurate Reporting of Relative Differences on Metabolic Status

**DOI:** 10.3390/molecules28052283

**Published:** 2023-03-01

**Authors:** Beatriz Vieira-da-Silva, Miguel A. R. B. Castanho

**Affiliations:** Instituto de Medicina Molecular, Faculdade de Medicina, Universidade de Lisboa, 1649-028 Lisboa, Portugal

**Keywords:** resazurin, cell viability, metabolic activity, fluorometric assay, UV–Vis assay, cytotoxicity

## Abstract

Cell viability and metabolic activity are ubiquitous parameters used in biochemistry, molecular biology, and biotechnological studies. Virtually all toxicology and pharmacological projects include at some point the evaluation of cell viability and/or metabolic activity. Among the methods used to address cell metabolic activity, resazurin reduction is probably the most common. At variance with resazurin, resorufin is intrinsically fluorescent, which simplifies its detection. Resazurin conversion to resorufin in the presence of cells is used as a reporter of metabolic activity of cells and can be detected by a simple fluorometric assay. UV–Vis absorbance is an alternative technique but is not as sensitive. In contrast to its wide empirical “black box” use, the chemical and cell biology fundamentals of the resazurin assay are underexplored. Resorufin is further converted to other species, which jeopardizes the linearity of the assays, and the interference of extracellular processes has to be accounted for when quantitative bioassays are aimed at. In this work, we revisit the fundamentals of metabolic activity assays based on the reduction of resazurin. Deviation to linearity both in calibration and kinetics, as well as the existence of competing reactions for resazurin and resorufin and their impact on the outcome of the assay, are addressed. In brief, fluorometric ratio assays using low resazurin concentrations obtained from data collected at short time intervals are proposed to ensure reliable conclusions.

## 1. Introduction

The use of molecules able to report the intracellular reducing environment through simple assays is extremely ubiquitous in biochemistry, molecular biology, and pharmacy laboratories. Molecules such as MTT, (3-(4,5-dimethylthiazol-2-yl)-2,5-diphenyltetrazolium bromide) tetrazolium, or resazurin (7-Hydroxy-3H-phenoxazin-3-one 10-oxide) are able to penetrate cells, undergo reduction in the intracellular space, and report the change in their oxidation state by colorimetric or fluorometric techniques. This process is used as a tool to draw conclusions regarding the metabolic activity of cells as the reducing environment can only be maintained through an operative intermediary metabolism.

When addressing methods to evaluate cytotoxicity, one should bear in mind that resazurin, like MTT, does not report the viability of cells as it does not report cell death, only metabolic activity. It may be used to compare the intrinsic reducing power of two different cell types, or the impact of a solute on a specific cell type in terms of metabolic dysfunction or impairment, but it cannot be used to unequivocally evaluate cell death. Direct cell death count by microscopic morphological alterations in cytoplasmic or nuclar structures, for instance, are methods appropriate to study cell death. Here, the focus is on resazurin reduction to study quantitative fold variations in metabolic activity.

The advantages and disadvantages of using resazurin over MTT have been reviewed before [1,2] and will be left out of the scope of this paper. Likewise, a broader comparison of different methods to address metabolic activity and viability is not within the realm of this study. Here, we will focus on the conditions that validate the resazurin assay as a reliable method to report on variations of cell activity. These conditions are frequently overlooked. Moreover, oversimplistic and erroneous assumptions are frequently adopted, explicitly or implicitly. For instance, the conversion of resazurin to resorufin is not a simple, irreversible reaction of two species, the reactant and the product. In addition, resorufin is frequently detected by fluorometry. At variance with electronic spectrophotometry/UV–Vis absorption, calibration in fluorometry is not straightforward [3].

## 2. Results

### 2.1. The Consecutive Reactions of Resazurin and Their Kinetics

**Scheme 1:**
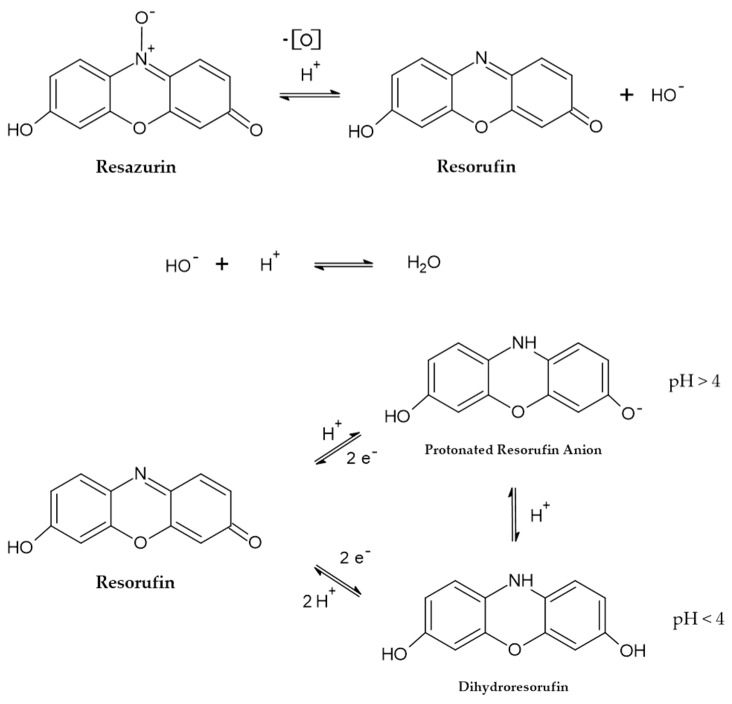
Reaction scheme of resazurin redox and acid-base reaction into resorufin. At different pHs further products are formed; protonated resorufin anion for pH > 4 and dihydroresorufin when pH < 4.

The details of resazurin redox and acid-base reaction were addressed thoroughly by voltammetry with a mercury electrode [4], which is a suitable model for biological redox reactions. The basics of resazurin redox reaction at different pHs is represented in reaction Scheme 1:

For the sake of simplicity, we will represent this reaction scheme by:

(1) $$A\overset{k_{1}}{\to }B\overset{k_{2}}{\to }C$$

in which A is resazurin, B is resorufin, and C are the end products of degradation (mainly protonated resorufin anion when pH > 4); the reaction rate constant *k*_1_ is dependent on the intrinsic rate of the reaction and the quantity of reducing species in the environment (NAD(P)H) or cytochrome c oxidase in the case of cells [1]. By approximation, *k_1_* can then be decomposed in:

(2) $$k_{1}=k_{1}^{\prime }\cdot \left[C_{v}\right]$$

$k_{1}^{\prime }$ is the intrinsic rate constant and $\left[C_{v}\right]$ is the number of viable cells per unit volume.

The fluorescence quantum yield of B at or close to λ_exc_ = 570 nm is much higher than the one of A or C, so the kinetics of the reaction can be followed by the time evolution of the intensity of fluorescence emission of B, $I_{f,\;B}\left(t\right)$. $I_{f,\;B}\left(t\right)$ is proportional to [B] in circumstances in which the inner filter effect [5] is not significant and interfering processes such as aggregation are not operative.

The time dependence of [B], [B](*t*), can be quantitatively established using the iterative method at short fixed-time intervals, ∆*t* [6]:

(3) $$\left[B\right]\left(t+∆t\right)=\left[k_{1}\left[A\right]\left(t\right)-k_{2}\left[B\right]\left(t\right)\right]\cdot ∆t+\left[B\right]\left(t\right)$$

The following condition applies:

(4) [B](t=0)=0

Using $I_{f,\;B}\left(t\right)$ instead of [B](*t*):

(5) $$I_{f,B}\left(t\right)=k\cdot \left[B\right]\left(t\right)$$

(6) $$I_{f,B}\left(t+∆t\right)=\left[k\cdot k_{1}\cdot \left[A\right]\left(t\right)-k_{2}I_{f,B}\left(t\right)\right]\cdot ∆t+I_{f,B}\left(t\right)$$

*k* is a proportionality constant dependent on the fluorescence quantum yield of B.

The slope of $I_{f,\;B}\left(t\right)$ at *t* = 0 is:

(7) $$\underset{t\overset{\;\;\;\;\;\;\;}{\to }0}{\lim}\left[\frac{I_{f,B}\left(t+∆t\right)}{∆t}\right]=k\cdot k_{1}\cdot \left[A\right]\left(t=0\right)$$

Comparing two distinct experimental conditions, hereafter named (a) and (b), such as absence and presence of a toxic solute, for instance, the slope ratio is:

(8) $$\frac{slope_{\left(a\right)}}{slope_{\left(b\right)}}=\frac{k_{1\;\left(a\right)}}{k_{1\;\left(b\right)}}=\frac{k_{1\;\left(a\right)}^{\prime }{\left[C_{v}\right]}_{\left(a\right)}}{k_{1\;\left(b\right)}^{\prime }{\left[C_{v}\right]}_{\left(b\right)}}$$

assuming *k* and [A](*t* = 0) are equal in the two conditions, (a) and (b). Additionally, assuming that $k_{1}^{\prime }$ is invariant between conditions (a) and (b):

(9) $$\frac{slope_{\left(a\right)}}{slope_{\left(b\right)}}=\frac{{\left[C_{v}\right]}_{\left(a\right)}}{{\left[C_{v}\right]}_{\left(b\right)}}$$

I.e., variations in the number of viable cells can be directly measured from the slope of the fluorometric kinetics monitoring of resorufin formation. In another type of experimental design, when comparing cells from different tissues at the same density, the slopes ratio corresponds to the ratio of the intrinsic reducing power of the two cell types $k_{1\;\left(a\right)}^{\prime }/k_{1\;\left(b\right)}^{\prime }$.

Slopes can be replaced by $I_{f,B}\left(t\right)$ measurements at a fixed time point, *t_x_*, as long as *t_x_* is short:

(10) $$\frac{I_{f,B}{\left(t_{x}\right)}_{\left(a\right)}}{I_{f,B}{\left(t_{x}\right)}_{\left(b\right)}}\approx \frac{{\left[C_{v}\right]}_{\left(a\right)}}{{\left[C_{v}\right]}_{\left(b\right)}}$$

This equation and its deduction constitute a quantitative validation to the assumptions qualitatively performed in some critical appraisals of the resazurin assay by others [7,8].

### 2.2. Assumptions and Limitations

Equation (10) is important as it establishes the theoretical framework for a truly quantitative deployment of the resazurin reduction assay to draw conclusions on variations of cell metabolic activity. Nonetheless, several assumptions were made while deriving it. These assumptions are practical limitations that cannot be overlooked as they need to be experimentally checked when applying the method.

#### 2.2.1. All Living Cells Have Equal Reducing Power and Non-Cellular Conversion of A to B Is Non-Significant

In practice, assuming Equation (2) is valid implies that viable cells are homogenous in their reducing power, and conversion of A to B caused by agents other than cells is non-significant. A can be converted to B by cells through electron transfer from NAD(P)H or by substituting molecular oxygen as an electron acceptor [1]. Either way, it reflects the activity of live cells.

In contrast, reduction may also occur through non-cellular electron donors, which may compromise the validity of Equation (4), therefore making Equation (10) not applicable. Although the resazurin assay is not as susceptible as similar assays, such as MTT, to small molecule interference, this is a possibility [2,9]. Nevertheless, a specific study using *E. faecalis* found that, under anaerobic conditions, resazurin conversion to resorufin can only happen intracellularly, while resorufin reduction can happen both intracellularly and extracellularly [10].

#### 2.2.2. Instrumental Factors in Fluorimetry

Equation (5) is only valid in specific conditions, which are critically dependent on instrumental (“geometric”) factors. The linear dependence of the fluorescence emission intensity on the concentration of a given solute requires the absence of significant inner filter effects [3,5], i.e., solute concentration sufficiently high to attenuate the intensity of the excitation bean at the focus point from where fluorescence emission is collected. Moreover, high concentrations of B may lead to binding to interfering agents with alteration of the fluorescence quantum yield. B concentrations should be kept as low as possible so that the sensitivity at the specific experimental conditions used is not jeopardized. Furthermore, A is associated with cytotoxic effects [1,9], which constitutes further reason to use as low a resazurin concentration as possible in an experiment.

It should also be stressed that fluorescence signals are not registered by fluorometers relative to a blank sample signal, at variance with UV–Vis absorption. Therefore, the fluorescence intensity recorded is specific to each apparatus at any given experimental session. This means that *k*, in Equation (5), is specific to a certain batch of samples, assayed in a specific spectrofluorometer, for a specific experimental session. While UV–Vis absorption could be used in alternative to fluorimetry because absorbance is also proportional to solute concentration, without having this limitation the sensitivity of the technique is much lower, therefore demanding higher concentrations of resazurin.

#### 2.2.3. Only $\left[C_{v}\right]$ Varies While Other Factors Are Constant When Comparing Conditions (a) and (b)

While comparing the two different experimental conditions (a) and (b), such as the absence and the presence of a given toxic solute, Equation (10) only applies if $\left[C_{v}\right]$ is the only variable. Changes in the reduction power of the cells or differential interference from other solutes renders *k* different in conditions (a) and (b). The consequences are Equations (8)–(10) are no longer valid and, therefore, the resazurin assay will lead to biased results.

#### 2.2.4. Viability vs. Metabolic Activity

Overall, one should bear in mind that although sometimes abusively referred to evaluate cell viability, the resazurin reduction assay, in fact, assesses metabolic activity. Under certain conditions (e.g., some bacteria-forming biofilms), cells having very low metabolic activity may be mistakenly taken for non-viable cells. Proper controls are needed to assure that there is sufficient metabolic activity to unequivocally distinguish viable from non-viable cells.

### 2.3. Practical Examples from Literature

The experimental conditions used to implement the resazurin assay and subsequent data analysis are frequently based on empirics and chemical intuition. Although thorough studies are scarce but well-interpreted [7,8], they miss detailed quantitative analyses. Application of the data analysis methodology developed in Section 2.1 enables more robust conclusions and further proof of the validity of the procedure.

#### 2.3.1. Comparing the Reducing Power of Different Types of Cells

**Figure 1:**
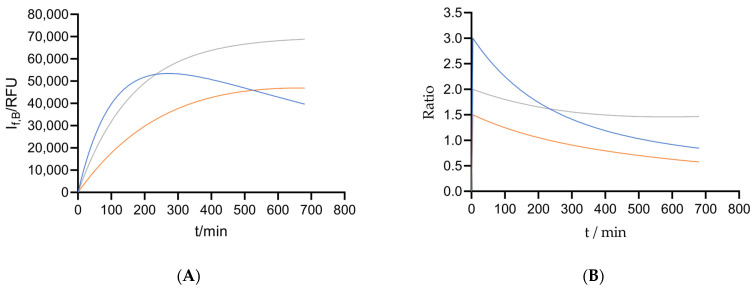
Fitting curves of the data presented by Uzarski et al. [7] on the reducing power of three different cell lines (panel **A**-TK188 fibroblasts, grey; Distal MDCK cells, blue; Proximal RPTE cells, orange) and ratio of the fitted curves obtained with any two out of the three cell lines (panel **B**-MDCK/RPTE, blue; MDCK/TK188, orange; TK188/RPTE, grey). For the fitted curves in panel A, $k_{2}/k_{1}$ was 0.11, 0.20 and 0.00 for MDCK, RPTE and TK188, respectively. [A](*t* = 0) was set equal for the three cell lines. Aditional data can be found in reference [7].

The data presented by Uzarski et al. [7] on the reducing power of three different cell lines, immortalized human renal fibrotic fibroblasts (TK 188), distal Madin–Darby canine kidney cells (MDCK), and proximal primary renal tubule epithelial cells (RPTE), can be fit by Equation (6) (Figure 1A). The ratio obtained with any two of the three cell lines, assuming there is an equal number of cells, is the estimated ratio of $k_{1}^{\prime }$, the intrinsic reducing power of the cells.

The intrinsic power of reducing resazurin is different in the three cell lines (Figure 1B) and the fate of resorufin is quite different as reflected by $k_{2}$ and denoted by the curves on Figure 1A after there is a decrease of the fluorescence intensity in MDCK cells and the formation of a plateau regarding the other two cell lines.

The ratio of $I_{f,B}\left(t\right)$ (Figure 1B) is clearly dependent on *t* for each pair of the three cell lines. The ratio at very short times (*t* ≈ 0) shows that MCCK cells are three-fold more reducing than RPTE cells but only 1.5-fold more than TK188, which is twice more reducing than RPTE. The relative scale of reducing power is thus MDCK > (1.5-fold) TK188 > (2-fold) RPTE.

It should be stressed that at longer times (*t* >> 0), $I_{f,B}\left(t\right)$ ratios vary greatly and may even fall below one. Therefore, when fluorescence intensities are not measured at sufficiently short intervals, the relative reducing power may be completely biased. This happens mainly in cases in which $I_{f,B}\left(t\right)$ curves have a significant descendent curve at long *t*, as seen in Figure 1A for the MDCK cells.

#### 2.3.2. Cytotoxicity: The Effect of Solutes on Cell Metabolic Activity

**Figure 2:**
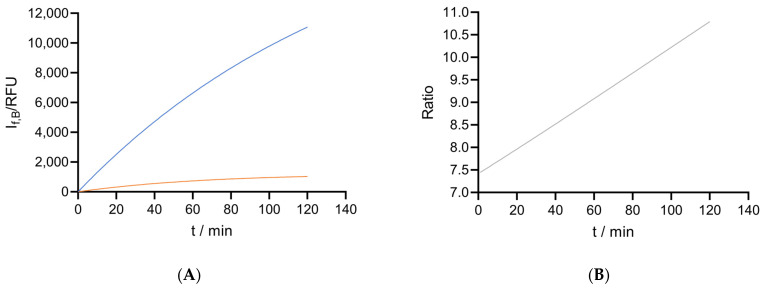
Fitting curves of the data presented by Lavogina et al. [8] on the metabolic activity of HeLa cells in the absence (blue) and presence of 10 μM doxorubicin (orange) (panel **A**) and ratio of the two fitted curves (panel **B**). For the fitted curves in (panel **A**), $k_{2}/k_{1}$ was 14.4 and 0.00 for the sample with and without doxorubicin, respectively. [A](*t* = 0) was set equal in both conditions.

Lavogina et al. [8] determined the kinetics of the formation of resorufin by HeLa cells in the absence and presence of 10µM doxorubicin, among other experimental data. Equation (7) was fit to the data (Figure 2A). The drop in the number of viable cells caused by doxorubicin was 7.4-fold, as shown by Figure 2B at *t* ≈ 0.

The examples of Figure 1 and Figure 2 demonstrate how a quantitative analysis over the resazurin assay can be performed to draw conclusions on the intrinsic reducing power of different cell lines or changes on cell cytotoxicity, depending on the experimental setup.

## 3. Conclusions

The resazurin reduction assay is ubiquitously used for its simplicity, low cost, and sensitivity. Nonetheless, its use up to now has relied on empirics and chemical intuition rather than on solid biochemical knowledge. We have derived the equations describing the kinetics of resorufin production and consumption and related the kinetic parameters to experimental conditions. The approach was validated by application of published data by independent laboratories. Such a quantitative approach opens new avenues to bridge chemistry, biology, toxicology, and pharmaceutical science into consensual and comparable applications of the resazurin assay.

It should be stressed that the nature of cells in culture and the variability of the levels of markers of cell viability among the individual cells in a population was not taken into account but may be relevant for more detailed expert approaches when viability is considered. In this case, specific approaches to cell viability, such as directly counting cells, could be considered. The use of multiplexed orthogonal methods to estimate the number of viable cells can be considered for more robust approaches [9].

### Good Practice in MTT and Resazurin Assay

The practical implications of our work for the end user can be condensed in a set of good practice rules. From a user’s perspective, the relevance of our work is the identification of the experimental conditions that make resazurin assay reliable to evaluate the toxicity of a certain compound on a given cell type or to evaluate the intrinsic reducing power of any two cell types. The latter challenge is similar to the evaluation of two different growth conditions on a given cell type.

Practical tips for a meaningful application of resazurin, or any other reducing power-based viability assay, are as follows:

(1) Use as low a resazurin concentration as possible because toxicity issues and technical spectroscopic biases may arise. The minimal concentration you can use depends on the signal-to-noise ratio of your detection system.
(2) Test for the reducing power of the medium in the absence of cells. Make sure it is nonsignificant when compared to cell reducing power in the absence of any toxic agents.
(3) Use data obtained at the shortest time interval possible, i.e., resazurin reduction data (formation of resorufin) should be registered as soon as possible after resazurin addition to cells, provided that the read-out is above the detection limit of the experimental setup you are using.
(4) Observance of the three previous rules allows quantitative ratiometric analysis, i.e., the fold variation of the measured response (fluorescence intensity or UV-Vis. absorption) is the fold variation of metabolic activity or intrinsic reducing power of cells.

## Data Availability

Not applicable.
